# Isolation and Identification of Secondary Metabolites from the Aerial Parts of *Stachys lavandulifolia *Vahl.

**Published:** 2017

**Authors:** Seyed Ebrahim Sajjadi, Seyed Mustafa Ghanadian, Mohammad Rabbani, Fateme Tahmasbi

**Affiliations:** a *Department of Pharmacognosy, School of Pharmacy and Pharmaceutical Sciences, Isfahan University of Medical Sciences, Isfahan, Iran. *; b *Department of Pharmacology and Toxicology, School of Pharmacy and Pharmaceutical Sciences, Isfahan University of Medical Sciences, Isfahan, Iran. *; c *Isfahan Pharmaceutical Sciences Research Center, School of Pharmacy and Pharmaceutical Sciences, Isfahan University of Medical Sciences, Isfahan, Iran.*

**Keywords:** *Stachys lavandulifolia*, labdan diterpenoid, flavonoid, monoterpen lacton, Lamiaceae

## Abstract

*Stachys lavandulifolia* Vahl is an herbaceous wild plant native to Iran which is traditionally used in Iranian folk medicine as a mild sedative tea for reducing anxiety and for treatment of gastrointestinal disorders. Our previous study on ethyl acetate extract of *S. lavandulifolia* proved anti-anxiolytic activity and so the present study was designed to determine chemical components of this biologically active fraction. The extract was prepared using maceration method. Column chromatography and medium pressure liquid chromatography (MPLC) was used respectively to separate the fractions. Finally, some evaluated fractions were used for high pressure liquid (HPLC) and peak shaving recycle technique to achieve more purification. Separated compounds were determined using NMR analysis and mass spectroscopy. Six compounds have been isolated from ethylacetate extract of aerial parts of *S. lavandulifolia* including four flavonoids (apigenin, kumatakenin, penduletin and 4', 7-dihyroxy- 3, 5, 6-trimethoxy flavon), a labdan diterpenoid (labda-13-en-8, 15-diol), and an iridoid.

## Introduction

The genus *Stachys* is one of the largest genera in the flowering plant family of Lamiaceae with about three hundred species distributed in Europe, Asia, Africa, Australasia, and North America(1).

*Stachys lavandulifolia* Vahl is an herbaceous wild plant native to Iran (2) which is used in Iranian folk medicine as a mild sedative tea for reducing anxiety and for treatment of gastrointestinal disorders (3).

Previous study on hydroalcoholic, polyphenolic, and boiled extracts of *S. lavandifolia *demonstrated their analgesic effect on animal models (4). In another study, antimicrobial and antioxidant activities of the essential oil of *S. lavandulifolia* were observed (5). Our previous study on *S. lavandulifolia* hydroalcoholic extract by the elevated plus-maze (EPM) model of anxiety in mice showed anxiolytic effect at therapeutically acceptable doses (6). Further study on petroleum ether, ethyl acetate, butanol and aqueous fractions on spontaneous motor activity and elevated plus-maze behavior in mice showed interesting anxiolytic properties for ethyl acetate fraction (7) and thus, we decided to investigate the main chemical constituents responsible for anxiolytic effects of ethyl acetate extract of *S. lavandulifolia.*

In previous researches on polar fractions of methanol extract of this plant, lavandulifolioside A, lavandulifolioside B, verbascoside, leucosceptoside A, 5-O-allopyranosyloxy-aucubin together with three phenylethanoid glycosides were reported (8). The essential oil of aerial part of *S. lavandulifolia* was also analyzed by GC/ MS method and germacrene-D (13.2%), β-phellandrene (12.7%), β-pinene (10.2%), myrcene (9.4%), α-pinene (8.4%) as well as Z-β-ocimene (5.8%) reported as the main components of the essential oil (9).

**Figure 1:**
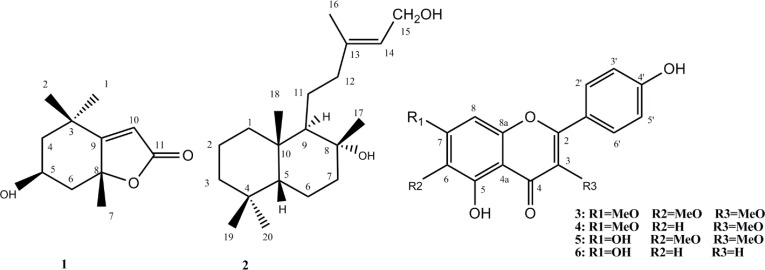
Chemical structures of compounds 1-6

## Experimental

*General*

NMR spectra were recorded on a Bruker Avance AV 400, using methanol-d4 as solvent. ESI-MS spectra were measured in positive and negative mode on Shimadzu 2010EV LC-MS system (Shimadzu, Japan). Column chromatography runs were performed using Silica gel, 63-200µm (Merck) and polyamide SC6 (Macherey- Nagel, Duren, Germany). HPTLC was performed on silica gel GF_254_ plates (Merck, Darmstadt, Germany). Plates were developed by Cerium sulphate (1 g in 5% H_2_SO_4_) or natural product reagent (1% methanolic diphenyl-boric acid-ethanolamine) and visualized by UV-fluorescent colours at 254 /366 nm UV lamps. Recycle HPLC was done on a modified Waters HPLC apparatus (Waters Assoc., Milford, MA, USA) at 250 nm using silica gel column (YMC-Pack SIL, 250 × 20 mm, YMC Co., Kyoto, Japan). 

*Plant material*

*S. lavandulifulia* was collected from Shahrekord city in Chaharmahal va Bakhtiari Province in the west of Iran. The plant was identified by the Department of Biology, Faculty of Science, University of Isfahan and also a voucher specimen (No. 1113) was deposited in the herbarium of the Isfahan Faculty of Pharmacy, Isfahan University of Medical Sciences, Isfahan, Iran.

*Extraction and isolation*

Following with our previous study which ethyl acetate fraction of *S. lavadulifolia* showed anxiolytic effect based on spontaneous motor activity and elevated plus-maze behavior in Syrian mice (7), in the same order ethyl acetate extract of the plant (5 kg) was obtained. Filtration and vacuum evaporation resulted in a green mass (254 g). After partitioning between hexane and methanol in separatory funnel, lower defatted methanol fraction (56 g) was concentrated and column chromatographed on silica gel (63-200 µm, 400 g) using hexane/acetone, with increasing polarity (5→50 %) to afford five fractions (F1-F5). The fraction of F2 which was eluted by hexane: acetone (9:1) was applied on HPLC using chloroform: methanol (92:8) as solvent and yielded F2c2 (1) as a pure iridoid. F3 eluted by hexane: acetone (8:2) precipitated as yellowish crystals which was more purified by recycle HPLC using chloroform: methanol (90:10) to yield F3b1 (2) and F3b2 (4) as a labdane diterpene and a methoxylated flavonoid, respectively. Finally, F4 eluted with hexane: acetone (7:3) and F5 eluted with hexane: acetone (5:5) were applied on recycle HPLC using chloroform: methanol (85:15) and F4f2b (3), F5c2a (5), and F5e (6) with flavonoid structure were isolated.

*Compound 1*

Amorphous white powder, MW (g/mol): 196; yield: 0.00016%; ^1^H-NMR (CDCI_3_, 400 MHz): δ_H_ 5.71(s, 1H, H-10), 4.24 (m, 1H, H-5), 2.45 (1H, bd, J=14.1, H-4b), 1.93 (1H, bd, J=14.1, H-4a), 1.80 (s, 3H, H-7), 1.52 (1H, m, H-6b), 1.49 (s, 3H, H-1), 1.35(1H, m, H-6a), 1.30 (s, 3H, H-2). ^13^C-NMR data (CDCl_3_, 100 MHz): 30.6 (C-1), 26.9 (C-2), 35.9 (C-3), 47.3 (C-4), 66.8 (C-5), 45.6 (C-6), 26.5 (C-7), 86.8 (C-8), 173.8 (C-9), 112.9 (C-10), 182.6 (C-11). EIMS *m/z* 196 [M], 178, 163, 152, 140, 125, 111, 95, 81, 69, 57.

*Compound 2*

Amorphous white powder, MW (g/mol): 308; yield: 0.00015%; ^1^H-NMR (CDCI_3_, 400 MHz): δ_H_ 5.38 (t, 1H, J=6.8Hz, H-14), 4.09 (d, 2H, J=6.8Hz, H-15), 1.70 (s, 3H, H-16), 1.14 (s, 3H, H-17), 0.90 (s, 3H, H-18), 0.86 (s, 3H, H-19), 0.84 (s, 3H, H-20). ^13^C-NMR data (CDCl_3_, 100 MHz): 41.2 (C-1), 19.5 (C-2), 44.3 (C-3), 34.2 (C-4), 57.5 (C-5), 21.5 (C-6), 43.2 (C-7), 74.9 (C-8), 62.5 (C-9), 40.4 (C-10), 25.2 (C-11), 45.0 (C-12), 140.9 (C-13), 124.1 (C-14), 59.4 (C-15), 16.4 (C-16), 33.9 (C-17), 16.1 (C-18), 21.9 (C-19), 23.8 (C-20). Positive ESIMS *m/z* 331 [M+Na]^+^, 309 [M+H]^ +^, 306, 290, 275.

*Compound 3*

Amorphous yellow powder, MW (g/mol): 344; yield: 0.0022%; ^1^H-NMR (CDCI_3_, 400 MHz): δ_H_ 8.05 (d, 2H, J=8.8Hz, H-2', H-6'), 6.95 (d, 2H, J=8.8Hz, H-3', H-5'), 6.79 (s, 1H, H-8), 3.99 (s, 3H, OMe), 3.86 (s, 3H, OMe), 3.82 (s, 3H, OMe). ^13^C-NMR data (CDCl_3_, 100 MHz): 158.6 (C-2), 139.5 (C-3),180.3 (C-4), 107.3 (C-4a), 154.0 (C-5), 133.4 (C-6), 161.9 (C-7), 92.1 (C-8), 153.4 (C-8a), 122.5 (C-1'), 131.5 (C-2'), 116.6 (C-3'), 160.6 (C-4'), 116.6 (C-5'), 131.5 (C-6'), 60.6 (3-OMe), 61.1 (6-OMe), 57.0 (7-OMe). EIMS *m/z* 344 [M], 329, 207, 197, 181, 167, 158, 149, 131, 121, 93, 69, 57.

*Compound 4*

Amorphous yellow powder, MW (g/mol): 314; yield: 0.001%; ^1^H-NMR (CDCI_3_, 400 MHz): δ_H_ 8.05 (d, 2H, J=8.8Hz, H-2', H-6'), 6.95 (d, 2H, J=9.2Hz, H-3', H-5'), 6.66 (d, 1H, J=2.4 Hz, H-8), 6.37 (d, 1H, J=2.4 Hz, H-6), 3.91 (s, 3H, OMe), 3.81 (s, 3H, OMe). ^13^C-NMR data (CDCl_3_, 100 MHz): 158.0 (C-2), 138.2 (C-3), 184.1 (C-4), 105.0 (C-4a), 154.3 (C-5), 98.8 (C-6), 161.4 (C-7), 93.0 (C-8), 154.3 (C-8a), 122.5 (C-1'), 131.2 (C-2'), 116.4 (C-3'), 158.2 (C-4'), 116.4 (C-5'), 131.2 (C-6'), 60.5 (3-OMe), 56.3 (7-OMe). Negative ESIMS *m/z* 313 [M-1]^-^, 298, 269, 255, 227, 187, 166, 154, 136.

*Compound 5*

Amorphous yellow powder, MW (g/mol): 330; yield: 0.0035%; ^1^H-NMR (CDCI_3_, 400 MHz): δ_H_ 7.89 (d, 2H, J=8.8Hz, H-2', H-6'), 6.83 (d, 2H, J=8.8Hz, H-3', H-5'), 6.41 (s, 1H, H-8), 3.78 (s, 3H, OMe), 3.67 (s, 3H, OMe). ^13^C-NMR data (CDCl_3_, 100 MHz): 153.7 (C-2), 139.1 (C-3), 180.3 (C-4), 106.3 (C-4a), 153.8 (C-5), 132.6 (C-6), 158.8 (C-7), 95.0 (C-8), 158.2 (C-8a), 122.5 (C-1'), 131.4 (C-2'), 116.5 (C-3'), 161.8 (C-4'), 116.5 (C-5'), 131.4 (C-6'), 60.6 (3-OMe), 61.0 (6-OMe). Negative ESIMS *m/z* 329 [M-1]^-^, 314, 299, 271, 227, 187, 166, 154, 125, 111.

*Compound 6*

Amorphous yellow powder, MW (g/mol): 269; yield: 0.0065%; ^1^H-NMR (CDCI_3_, 400 MHz): δ_H_ 7.88 (s, 1H,OH), 7.73 (d, 2H, J=8.8Hz, H-2', H-6'), 6.85 (d, 2H, J=8.8Hz, H-3', H-5'), 6.49 (s, 1H, H-3), 6.37 (d,1H, J=2Hz, H-8), 6.14 (d,1H, J=2Hz, H-6). ^13^C-NMR data (CDCl_3_, 100 MHz): 163.8 (C-2), 104.4 (C-3), 183.2 (C-4), 106.5 (C-4a), 163.2 (C-5), 100.5 (C-6), 166.4 (C-7), 95.3 (C-8), 159.0 (C-8a), 122.8 (C-1'), 129.4 (C-2'), 117.3 (C-3'), 163.3 (C-4'), 117.3 (C-5'), 122.8 (C-6'). Negative ESIMS m/z 269 [M-1]^-^, 257, 227, 195, 181, 173, 155, 129, 110.

## Results and discussion

The ^13^C NMR and ^1^H-NMR spectra of compound 1 indicated signals of three singlet methyl groups, two methylene, an oximethine, two quaternary carbons which one of them was oxygenated, a tri-substituted olefin bond and a lactone carbonyl with EIMS *m/z* 196 which resembled with those of a C11 iridoid named loliolide (10). The iridoids and secoiridoids form a large group of plant constituents that are found usually. They are mostly 4, 7-dimethylcyclopentapyran. But in the case of loliolide (C11 iridoid) and its related structures like actinidiolide and aeginetolide their biosynthesis pathway is different from usual C-10 iridoids and secoiridoids. They are generated in variety of plants from photo-oxygenation and degredation of carotenoids (11-12). 

Compound 2 was obtained as a white powder. ^13^C-NMR data in addition to positive ESIMS at *m/z* 331 [M+Na]^ + ^corresponded to the formula C_20_H_36_O_2_. ^1^H NMR spectrum showed signals attributed to five singlet methyls δ_H_ 1.70, 1.14, 0.90, 0.86, and 0.84. Further signals in ^1^H NMR spectrum at δ_H_ 5.38 (t, 1H, J=6.8Hz, H-14) and 4.09 (d, 2H, J=6.8Hz, H-15) indicated the presence of a tri-substituted olefin bond and an external oxymethylen group. The ^13^C-NMR and DEPT spectra demonstrated twenty carbons comprised of five methyls, eight aliphatic methylens (one oxygenated), two aliphatic methines, two aliphatic quternary carbons (one oxygenated), one olefinic methine, and one quaternery olefinic carbon. Three degrees of unsaturation, and one olefinic bond suggested two rings in the structure. Comparing these characteristic signals with compound 6 isolated from *Leonurus heterophyllus* by Hung and coworkers (14) and compound 8 isolated from *Leonurus heterophyllus *by Giang and coworkers (13) and using HMBC spectra determined structure of compound 2 as labda-13-ene-8, 15-diol.

**Figure 2:**
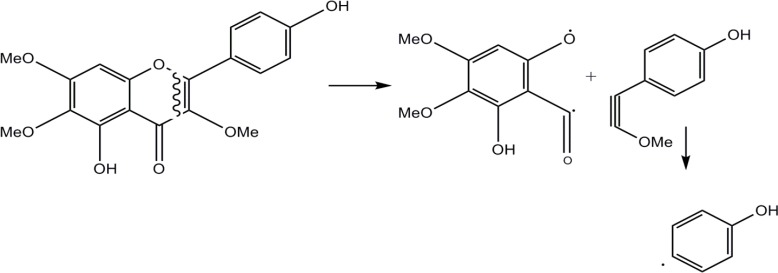
EI-Mass fragmentation pattern of compound 3. Cleavage of ring C by retro-Diels-Alder mechanism led to A (*m/z* 197), B (*m/z* 149), and C (*m/z* 93) ions

Compound 3 was isolated as a yellowish powder with orange fluorescence reaction to Natural Product reagent (Diphenylboric acid 2-aminoethyl ester 1%). Molecular formula was determined by NMR data and negative EIMS m/z 344 [M] as C_18_H_16_O_7_. Eleven degrees of unsaturation, one carbonyl carbon, and seven olefin bands indicated three rings in the structure. The ^1^H-NMR spectrum showed a singlet olefin proton at δ 6.79 (s, 1H) described to H-8. *Ortho*-coupled proton signals( each two protons) at δ 8.05 (d, 2H, J=8.8Hz) and 6.95 (d, 2H, J=8.8Hz) were corresponded to H-2’,6' and H-3', 5'. Three singlet methoxy signals at δ_H_ 3.99 (δ_C_ 61.1), 3.86 (δ_C_ 60.6), 3.82 (δ_C_ 57.0) were located on C-3, C-6 and C-7, respectively based on long range HMBC correlation and cleavage pattern of compound in EI-Mass by retro-Diels-Alder mechanism including A (*m/z* 197), B (*m/z* 149) and C (*m/z* 93) ions. All of these data defined the structure of 3 as 3, 6, 7-trimethoxy-4',5-dihydroxyflavone named as penduletin which was in agreement with literature data (15). Figure 2 shows the fragmentation pattern of this compound by cleavage of ring C via retro-Diels-Alder mechanism.

Compound 4 assigned the molecular formula of C17H14O6 based on negative pseudo-molecular ion [M - H]^-^ at *m/z* 313 and ^13^C-NMR (BB and DEPT). ^1^H & ^13^C-NMR spectral data cleared that compound 4 and 3 have similar structures but differed in lacking methoxy group at C-6. Comparison of the spectral data with literature data determined the structure as 7,3-dimethoxy-4',5-dihydroxyflavone named as Kumatakenin (16). Similarly, NMR spectral data cleared that compounds 5 and 3 resembled each other, except for the 7-O-hydroxyl instead of 7-O-methoxyl group which was confirmed by pseudo-molecular ESI Mass ion [M - H]^-^ at *m/z* 329. It was defined as 6, 3-dimethoxy-4', 5-dihydroxyflavone (17). Compound 6 was proved to be 4', 5, 7-trihydroxyflavone named as apigenin (17).

Many species of the genus *Stachys* have been investigated and different kinds of secondary metabolites have been isolated, mainly flavonoids, iridoids and terpenoids from the aerial parts and the roots (18). In this investigation, an iridioid, a labdane type diterpene, three methoxylated flavonol derivatives, and a flavone were isolated.

Previous phytochemical studies showed presence of several types of flavonoids in *Stachys* genus. Some *Stachys* species are rich in flavonoids, for example 24 flavonoids were identified in *S. aegyptica* and 9 in *S. ionica *(19, 20). However in this study, three methoxylated flavonol derivatives, and a flavone were isolated from *S.lavandulifolia*. Compound 3 with trivial name of kumatakenin, previously was isolated from *Ballota hirsuta*, *Eupatorium illitum*, *Achillea kotschya,* and *Baccharis petiolata*. The flavonoids from *Eupatorium illitum* have been presented antiproliferative activity (22). Compound 4 with trivial name of penduletin, was previously isolated from *Achillea nobilis, Isocoma tenuisecta* and *Betola nigra* (21). Pendulentin has showed antiproliferative property (23) and strong activity against enterovirus (24). Compound 5, was previously reported from *Achillea kotschya, Achillea nobilis, *and some other genera of Asteraceae and apigenin is also determind in many *Stachys* species 21).

The diterpenoids isolated from different species of *Stachys* are structurally different with labdane, kauran, clerodane, and abietan skeletone. Kauran diterpenoids are present in *S. silvatica*, *S. lanata*, and *S. sprumeri* and also *S. recta*, neo-clerodan derivatives are found in *S. annua*, *S. rosea,* and *S. recta*. An abietan derivative in *S. officinalise* and diterpenoids with labdane skeleton were also obtained from *S. mucronata*,* S. plumasa*, and *S. menthifolia *(18, 25). The labdane diterpenoid which is isolated in this study was also previously reported from *S.menthifolia*, *Leonurus heterophyllus*, and *Cistus creticus* (11, 18).

## References

[1] Evans WC (1989). Trease and Evans’ Pharmacognosy.

[2] Mozaffarian V (1996). A Dictionary of Iranian Plant Names.

[3] Amin GH (1991). Popular Medicinal Plants of Iran.

[4] Hajhashemi V, Ghannadi A, Sedighifar S (2006). Analgesic and anti-inflammatory properties of the hydroalcoholic, polyphenolic and boiled extracts of Stachyslavandulifolia. Res. Pharm. Sci.

[5] Iscan G, Demirci B, Demirci F, Goger F, Kirimer N, Kose YBand Baser KHC (2012). Antimicrobial and antioxidant activities of Stachys lavandulifolia subsp lavandulifolia essential oil and its infusion. Nat. Prod. Com.

[6] Rabbani M, Sajjadi SE, Zarei HR (2003). Anxiolytic effects of Stachys lavandulifolia Vahl on the elevated plus-maze model of anxiety in mice. J. Ethnopharmacol.

[7] Rabbani M, Sajjadi SE, Jalali A (2005). Hydroalcohol extract and fractions of Stachys lavandulifolia Vahl: effects on spontaneous motor activity and elevated plus maze behavior. Phytother. Res.

[8] Delazar A, Delnavazi MR, Nahar L, Moghadam SB, Mojarab M, Gupta A, Williams AS, Mukhlesur Rahman M, Sarker SD (2011). Lavandulifolioside B: a new phenylethanoid glycoside from the aerial parts of Stachys lavandulifolia Vahl. Nat. Prod. Res.

[9] Javidnia K, Mojab F, Mojahedi SA (2004). Chemical constituents of the essential oil of Stachys lavandulifolia Vahl from Iran. Iran. J. Pharm. Res.

[10] Tanaka R, Matsunaga S (1989). Loliolide and olean-12-en-3β, 9α, 11α-triol from Euphorbia supina. Phytochem.

[11] Isoe S, Hyeon SB, Katsumura S, Sakan T (1972). Photo-oxygenation of carotenoids The absolute configuration of loliolide and dihydroactinidiolide. Tetrahedron Lett..

[12] Murai F, Tagawa M, Ohishi H (1992). Absolute structure of kiwiionoside as a precursor of loliolide and actinidiolide, from Actinidia chinensis. Planta Mmed.

[13] Hung TM, Luan TC, Vinh BT, Cuong TD, Min BS (2011). Labdane‐type diterpenoids from Leonurus heterophyllus and their cholinesterase inhibitory activity. Phytother. Res.

[14] Giang PM, Son PT, Matsunami K, Otsuka H (2005). New labdane-type diterpenoids from Leonurus heterophyllus Sw. Chem. Pharm. Bull.

[15] Rodriguez E, Carman NJ, Vander Velde G, McReynolds JH, Mabry TJ, Irwin MA, Geissman TA (1972). Methoxylated flavonoids from Artemisia. Phytochem.

[16] Calvert DJ, Cambie RC, Davis BR (1979). 13C NMR spectra of polymethoxy‐and methylenedioxy flavonols. Org. Magn. Resonance.

[17] Agrawal PK (1989). Carbon-13 NMR of Flavonoids.

[18] Tundis R, Peruzzi L, Menichini F (2014). Phytochemical and biological studies of Stachysspecies in relation to chemotaxonomy: A review. Phytochem.

[19] El-Ansari MA, Barron D, Abdalla MF, Saleh AM, Le Quere JL (1991). Flavonoid constituents of Stachys aegyptica. Phytochem.

[20] Meremeti A, Karioti A, Skaltsa H, Heilmann J, Sticher O (2004). Secondary metabolites from Stachys ionica. Biochem. Syst. Ecol.

[21] Harborne JB (1994). The Flavonoids, Advances in Research since 1980.

[22] Castillo QA, Triana J, Eiroa JL, Padron JM, Plata GB, Abel-Santos EV, Báez LA, Rodríguez DC, Jiménez MA, Pérez-Pujols MF (2015). Flavonoids from Eupatorium illitum and their antiprolifrative activities. Pharmacog. J.

[23] Moghaddam Gh, Ebrahimi SA, Rahbar-Roshandel N, Foroumadi A (2012). Antiprolifrative activity of flavonoids: influence of sesquentalmethoxylation state of the flavonoid structure. Phytother. Res.

[24] Zho QC, Wang Y, Liu YP, Zhang RQ, Li X, Su WH, Long F, Luo XD, Peng T (2011). Inhibition of enterovirus 71 replication by chrysosplentin and penduletin. Eur. J. Pharm. Sci.

[25] Piozzi F, Bruno M (2011). Diterpenoids from roots and aerial parts of the genus Stachys. Rec. Nat. Prod.

